# Tribological and Hygroscopic Behavior of Polybutylene Terephthalate/Acrylonitrile Styrene Acrylate (PBT/ASA) Nanocomposites with Graphene Nanofiller

**DOI:** 10.3390/polym16223149

**Published:** 2024-11-12

**Authors:** Pyoung-Chan Lee, Seo-Hwa Hong, Ji Taek Oh, Donghyeok Shin, Jae-Uk Jung, Youn Ki Ko, Jin Uk Ha, Myeong-Gi Kim, Beom-Gon Cho

**Affiliations:** 1 Chassis & Materials Research Laboratory, Korea Automotive Technology Institute, 303 Pungse-ro, Pungse-myeon, Dongnam-gu, Cheonan-si 31214, Chungcheongnam-do, Republic of Korea; pclee@katech.re.kr (P.-C.L.); shhong1@katech.re.kr (S.-H.H.); ykko@katech.re.kr (Y.K.K.); juha@katech.re.kr (J.U.H.); 2 R&D Center, BESTGRAPHENE Co., Ltd., Yeoju-si 12616, Gyeonggi-do, Republic of Korea; ojt3413@best-graphene.com; 3 R&D Center, WOOSUNG Advanced Materials Co., Ltd., Mungyeong-si 36990, Gyeongsangbuk-do, Republic of Korea; sdh@metapoly.co.kr; 4 R&D Planning Department, Nifco Korea Co., Ltd., Asan-si 31409, Chungcheongnam-do, Republic of Korea; jujung@nifco.co.kr; 5 Department of Polymer Science and Engineering, Kumoh National Institute of Technology, 61 Daehak-ro, Gumi-si 39177, Gyeongsangbuk-do, Republic of Korea

**Keywords:** polybutylene terephthalate (PBT), acrylonitrile styrene acrylate (ASA), nanocomposites, fogging, headlamps, graphene

## Abstract

Fogging in automotive headlamps is a significant issue that affects both aesthetics and functionality. This study investigates the use of graphene-based nanocomposites to mitigate fogging by enhancing the hygroscopic properties of polybutylene terephthalate/acrylonitrile styrene acrylate (PBT/ASA) composites commonly used in headlamps. The incorporation of functionalized graphene improved the tensile and flexural strength of the nanocomposites, though it led to a reduction in elongation and melt flow. Additionally, the solid lubrication properties and increased surface hardness of the graphene contributed to enhanced wear resistance. The presence of graphene in the nanocomposites also reduced moisture diffusion, lowering the rates of both hygroscopic and desorption when compared to commercial PBT/ASA composites. Furthermore, the nanocomposites exhibited a reduction in maximum moisture uptake. These improvements are expected to reduce the absolute humidity inside the headlamp, thereby effectively mitigating the fogging issue.

## 1. Introduction

With the increasing focus on enhancing vehicle performance, there is growing interest in safety and convenience technologies. Features such as parking assistance and collision avoidance systems are being widely implemented, and attention is also being given to design-related technologies that affect the vehicle’s exterior quality. Among these, the functionality of headlamps has become a crucial aspect. Headlamps are essential for illuminating a vehicle’s path in low-visibility conditions, while tail lamps serve as indicators and signal lamps. Recently, headlamps have evolved from simple lighting devices to design-forward safety features. Initially, headlamps used basic lighting sources but have transitioned through incandescent bulbs, high-intensity discharge (HID), and LEDs. With this progression, lamp designs have also become more complex, leading to the increased use of polymer composites due to their design flexibility [1,2,3,4,5,6,7,8,9,10]. Polymer composites are typically formed through injection molding, offering excellent moldability and the ability to achieve complex shapes. However, these materials are more susceptible to moisture compared to metals, which can degrade their properties over time, especially in humid environments [8,9,10].

In headlamps, the sealed environment causes temperature fluctuations during operation, leading to the absorption and release of moisture in plastic components, which alters the internal humidity and contributes to fogging. Moisture absorbed into polymer-based composites can infiltrate the material’s free volume and micropores created during the absorption/desorption process, resulting in various reversible or irreversible changes due to temperature variations [9,10,11,12,13,14,15,16,17]. Research into the hygroscopic behavior of polymer composites, which affects headlamp fogging, is critical, as this behavior directly impacts the primary function of headlamps: ensuring the driver’s visibility.

Most polymers are affected by moisture absorption, which influences their mechanical properties. Polymers with polar functional groups, such as polyamide and PBT, have particularly high moisture absorption rates. Two main methods have been explored to reduce moisture absorption in polar polymers. The first is blending or co-polymerizing with non-polar polymers, typically polyolefins. However, this can lead to a reduction in overall composite properties due to the lower performance of polyolefins. The second method involves developing composites by incorporating inorganic plate-like nanofillers, which alter the diffusion path of water molecules [9,10,11,12]. Such fillers, with sufficient aspect ratios, improve moisture barrier properties and can enhance mechanical and thermal characteristics [9,10,18]. For instance, our previous research demonstrated property improvements in PP nanocomposites using alkylated functionalized graphene as a nanofiller [10]. Additionally, Achagri et al. reported that graphene functionalized with alkylamines, when combined with PET and PBT, improved thermal and mechanical properties while imparting hydrophobicity.

In this study, we aimed to reduce fogging in automotive headlamps by controlling the moisture absorption and desorption behavior of PBT/ASA composites used for lens holders. The modified graphene as a plate-like filler was applied to hinder moisture diffusion, and alkylamine-functionalized graphene was synthesized to enhance its compatibility with the molecular structure of the PBT and ASA polymer chain. Furthermore, it was investigated that the lateral size of graphene affects the nanocomposite’s mechanical properties, hygroscopic control performance, and wear resistance.

## 2. Materials and Methods

### 2.1. Materials

To produce chemically modified graphene (CMG), graphene oxide solutions, BGO-S (lateral size 1–2 μm grade) and BGO-L (lateral size 5–10 μm grade), from BESTGRAPHENE Co., Ltd. (Yeoju-si, Republic of Korea) were used. Octadecan-1-amine (ODA, >98.0%) was obtained from TCI (Tokyo, Japan) and was used without further pretreatment. Polybutylene terephthalate (PBT) of PBT1084 grade was sourced from BLUE STAR Co. (Beijing, China), and acrylonitrile styrene acrylate (ASA) of LI911 grade was provided by LG Chem. Co. (Daejun, Republic of Korea). Furthermore, glass fiber of GF534A grade was supplied by JUSHI Co. (Tongxiang, China).

### 2.2. Preparation of Nanofiller

In this study, two types of nanofillers with different graphene lateral sizes were synthesized as follows. A 2 mg/mL aqueous solution was prepared by diluting 10 mg/mL of both BGO-S and BGO-L as starting materials. The 2 mg/mL aqueous solutions were subjected to ultrasonic treatment using an ultrasonic homogenizer for over 2 h to obtain graphene oxide aqueous dispersions. To improve the compatibility between graphene and PBT/ASA, surface alkylation of graphene was performed using octadecan-1-amine (ODA). Initially, 500 mL of a 0.1% BGO-S aqueous solution was prepared via ultrasonic treatment for at least 1.5 h using an ultrasonic homogenizer, and 10 g of ODA was added to 500 mL of ethanol. Subsequently, the ethanol solution, which had been stirred for 1 h at 60 °C, was mixed with the BGO-S aqueous solution and stirred again for 40 h at 90 °C to initiate a surface alkylation reaction.

The reaction product was cooled to room temperature, and the precipitate was collected by centrifugation, washed 5 to 8 times with ethanol, and filtered to obtain graphene. The precipitate was then dried in a convection oven at 60 °C for 4 h to yield functionalized graphene (CMG-RS) with a linearly alkylated surface, ensuring molecular compatibility with PBT/ASA. CMG-RL was synthesized using the same surface functionalization procedure, with ODA.

### 2.3. Preparation of Nanocomposites

The control samples, commercial PBT/ASA composites, were prepared using a 32 mm twin-screw extruder (L/D = 40/1). The compounding temperature was controlled between 220 °C and 250 °C, with a rotor speed set to 400 RPM. The composition of the PBT/ASA composites consisted of 51 wt.% of PBT, 15 wt.% of ASA, 30 wt.% of glass fiber, and 4 wt.% of other additives (such as antioxidants, lubricants, compatibilizers, etc.). The graphene content in the PBT/ASA nanocomposites was set at 0.05 wt.%, 0.10 wt.%, and 0.20 wt.%, with the corresponding weight of PBT/ASA (65.95 wt.%, 65.9 wt.%, and 65.8 wt.%, respectively) reduced by the weight of graphene. Graphene was incorporated into the mixture via a side feeder during the compounding process. Test specimens for moisture absorption and desorption experiments were injection-molded using a 100 x 100 mm mold, with a specimen thickness of 1 mm.

### 2.4. Characterization

The morphologies of the nanofillers (functionalized graphene) were investigated using scanning electron microscope (SEM, VEGA, TESCAN, Brno, Czech Republic) with 20 keV and SE mode. Meanwhile, Raman spectroscopy (Confotec MR350, SOL Instruments, Minsk, Belarus) was performed with a 532 nm CW DPSS laser to determine the chemical surface modification of nanofillers, and Fourier-transform infrared (FT-IR) spectroscopy was performed using a 670-IR spectrometer (PerkinElmer, Waltham, MA, USA) on nanofillers to investigate functionalization. The samples were analyzed in the transmittance mode over the scanning range of 800–4000 cm^−1^ with a resolution of 0.5 cm^−1^. Furthermore, dynamic light scattering (DLS, LITESIZER500, Anton Paar, Graz, Austria) and laser diffraction particle size analysis (LD-PSA, Microtrac S-3500, Microtrac, PA, USA) were used to analyze the lateral size of nanofillers. DLS equipment was used for CMG-RS and BGO-S, which have small particle sizes, and LD equipment was used for CMG-RL and BGO-L, which have large particle sizes.

The mechanical properties of the PBT/ASA nanocomposites were measured using a universal testing machine (UT-100F, MDTI Korea Co., Daejeon, Republic of Korea). Surface hardness was evaluated using a Shore D Durometer (GS-720, Teclock, Tokyo, Japan). The tribological behavior of the nanocomposites was assessed with a pin-on-disc tester (THT, Anton Paar, Graz, Austria) under testing conditions of 25 °C and 10 N, utilizing a 6 mm stainless-steel ball-type pin. The wear surfaces were examined using an optical microscope (VHX-700F, Keyence, Osaka, Japan).

Moisture absorption tests were conducted by cutting the specimens into 70 × 70 mm pieces. Prior to the moisture absorption experiments, the specimens were dried in an oven at 80 °C for approximately 48 h to measure the dry weight (W_dry_). The moisture absorption experiments were performed by placing the dried specimens in a constant temperature and humidity chamber (TH-G300, Jeio Tech, Daejeon, Republic of Korea) at varying temperatures (35 °C, 50 °C, and 65 °C) and 85% relative humidity (RH) for a specified time (*t*). At specific time intervals, the wet weight (*W_wet_*) of the specimens was recorded. The moisture absorption ($M_{a_{t}}$, %) was calculated using Equation (1), based on the *W_dry_* and *W_wet_* [11,12,13]. For each condition, 15 samples were tested to obtain the average moisture absorption values.

(1) $${Ma}_{t}=\frac{\left(W_{wet}-W_{dry}\right)}{W_{dry}}\times 100\;(\%)$$

After the moisture absorption experiments, the final weight (*W_w_*) of the specimens was measured, and moisture desorption was evaluated using a halogen moisture analyzer (HX204, Mettler Toledo, Columbus, OH, USA). The weight change (*W_d_*) was recorded at drying temperatures of 55 °C, 70 °C, and 85 °C. Moisture desorption ($M_{d_{t}}$) was calculated using the same method as moisture absorption (${Ma}_{t}$), following the formula below.

(2) $${Md}_{t}=\frac{\left(W_{w}-W_{d}\right)}{W_{w}}\times 100\;(\%)$$

To verify the moisture reduction effect of the developed materials, a headlamp was fabricated using PBT/ASA nanocomposites for the lens holder. The lens holder was manufactured through an injection molding process, referencing a mass-produced vehicle model. The injection molding conditions were as follows: injection temperature of 235–300 °C, mold temperature of 80 °C, injection pressure of 9 MPa, injection time of 4.3 s, holding pressure of 7 MPa, holding time of 8 s, and cooling time of 35 s. A test headlamp was produced by replacing the lens holder of the reference headlamp. A temperature and humidity sensor (SEK-SHT35, Sensirion AG, Stäfa, Switzerland) was installed within the headlamp to measure the absolute humidity inside the headlamp. The headlamp was preconditioned by placing it in an oven at 50 °C for 48 h, followed by 48 h in a 50 °C, 50% RH environment. The absolute humidity was measured by placing the headlamp in an oven set at 50 °C and applying a voltage of 13.5 V.

## 3. Results

### 3.1. Characterization of Nanofiller

**Figure 1:**
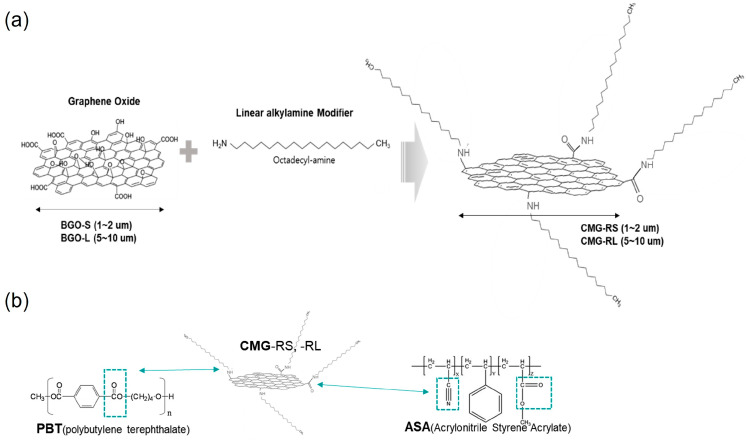
Scheme of the graphene surface modification process (**a**) and chemical interaction between CMG-RS, CMG-RL, and PBT/ASA in the nanocomposites (**b**).

Figure 1 illustrates a schematic diagram of the functionalization of graphene oxide through a chemical modification reaction with alkylamine. In this process, graphene oxide reacts with alkylamine, resulting in the attachment of linear alkyl hydrocarbon chains to the graphene oxide structure. This functionalization enhances the properties of graphene oxide, making it suitable for various applications in materials science and nanotechnology. In the described synthesis, BGO-S (small graphene oxide) has a lateral size of 1 to 2 μm, while BGO-L (large graphene oxide) has a lateral size of 5 to 10 μm. As a result, CMG-RS (chemically modified graphene by alkylation—small size) of 1 to 2 μm and CMG-RL (chemically modified graphene by alkylation—large size) of 5 to 10 μm can be created. The alkyl chains in the structure of CMG-RS and CMG-RL are expected to exhibit high compatibility with the main hydrocarbon chains of PBT and ASA. Furthermore, the -C-NH- and -CONH- groups formed at the connection points between the graphene surface and the alkyl chains in CMG-RS and CMG-RL are likely to form hydrogen bonds with the -COO- groups in PBT and the -COO- and -CN groups in ASA. This suggests that CMG-RS and CMG-RL will have excellent compatibility with the PBT/ASA matrix.

**Figure 2:**
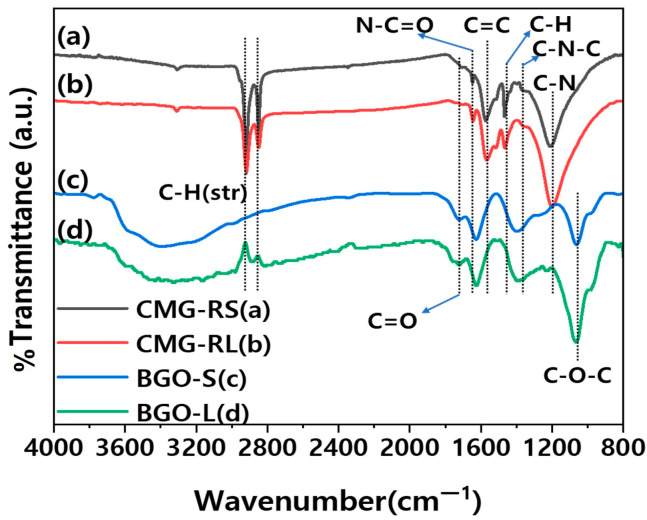
Fourier-transform infrared spectra of CMG-RS, CMG-RL, BGO-S, and BGO-L.

Figure 2 presents the FT-IR results for CMG-RS and CMG-RL, showing their transmittance spectra over the wavenumber range of 4000 to 800 cm^−1^. The successful functionalization reaction, which involved reduction, is evidenced by a significant reduction in the intensity of peaks related to the C=O (carbonyl group, 1725 cm^−1^) and C–O–C (epoxide group, 1055 cm^−1^) of graphene oxide (GO). Additionally, two peaks between 2850 and 2920 cm^−1^ and a strong peak at 1486 cm^−1^ were observed, corresponding to the linear hydrocarbon (–CH) of the alkyl group. Furthermore, peaks at 1080 cm^−1^ (attributed to C–N) and 1570 cm^−1^ (attributed to N–H) confirm that alkylation was successfully achieved [19]. The FT-IR analysis conclusively demonstrates the successful functionalization of graphene oxide with alkylamine, resulting in the incorporation of various functional groups such as carbonyl, amide, and aromatic groups. The similarity in peak positions between CMG-RS and CMG-RL suggests that the functionalization process is uniformly effective across different lateral sizes. However, the variations in peak intensities highlight differences in the degree of functionalization or the accessibility of these groups, likely influenced by the lateral size of the graphene sheets. Notably, the C-H (aliphatic) and C=C (aromatic) peak intensities are greater in CMG-RL than in CMG-RS, which can be attributed to the influence of the larger lateral size of CMG-RL.

**Figure 3:**
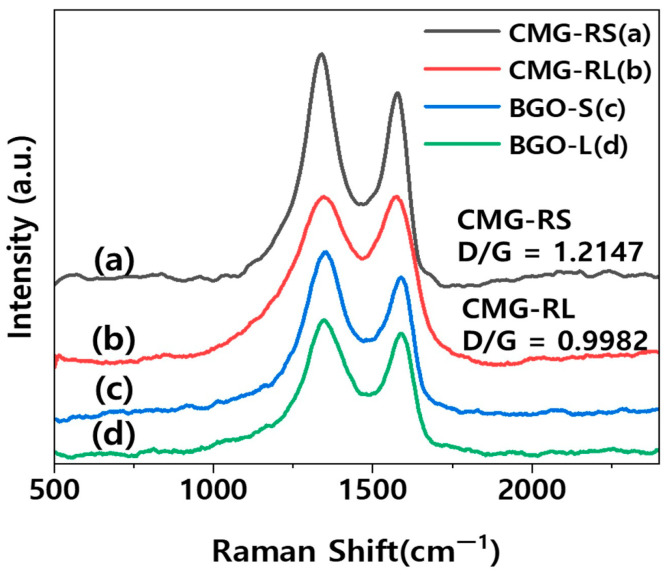
Raman spectra of CMG-RS, CMG-RL, BGO-S, and BGO-L.

**Figure 4:**
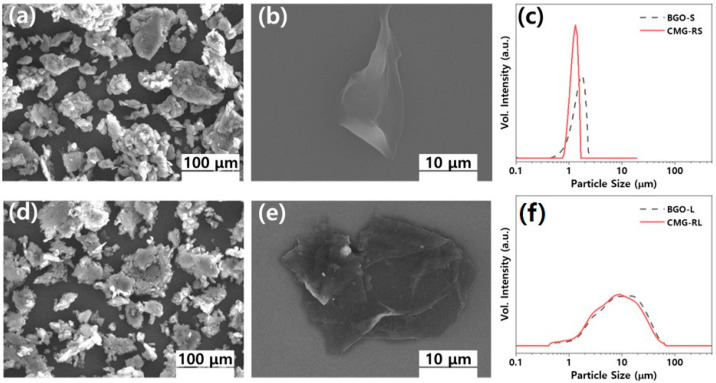
SEM images of CMG-RS (**a**,**b**) and CMG-RL (**d**,**e**) and particle size distribution of CMG-RS, BGO-S (**c**) and CMG-RL, BGO-L (**f**).

The structural characteristics of the as-synthesized CMG-RS and CMG-RL were identified through their Raman spectra. Figure 3 shows the Raman spectra of CMG-RS and CMG-RL produced by chemical surface modification, wherein the characteristic D band (near 1360 cm^−1^) and G band (near 1580 cm^−1^) peaks of graphene can be identified [18]. In the spectrum of CMG, the D/G ratio for CMG-RS was found to be 1.2147, while for CMG-RL, it was 0.9982. The higher D/G ratio for CMG-RS is due to its smaller lateral size, which results in more detectable edges compared to CMG-RL. Figure 4a,b,d,e display SEM images of dried CMG-RS and -RL powders, revealing granulated flakes with sizes ranging from 20 to 100 μm. The granulated structure of CMG-RS and -RL enhances workability during the polymer compounding process, improving the weighing, feeding, and premixing steps. Additionally, the alkyl chains inserted between graphene sheets prevent strong stacking caused by van der Waals forces and π–π interactions, allowing for easy separation into individual sheets. To test dispersion, the dried CMG-RS and -RL granules were mixed with xylene at 0.05 wt.% and shaken thoroughly. After applying one drop to a slide glass and drying, SEM observations confirmed that the granules easily separated into individual particles without aggregation, showing good dispersion. As shown in Figure 4 c,f, since CMG-RS and RL were synthesized using BGO-S and L, respectively, it can be confirmed that their sizes are similar. Furthermore, BGO-S shows 1.26 μm based on D50, and CMG-RS shows 0.912 μm. The sizes of BGO-L and CMG-RL are 8.30 μm and 7.77 μm based on D50, respectively (Figure 4f).

### 3.2. Characterization of Nanocomposites

**Figure 5:**
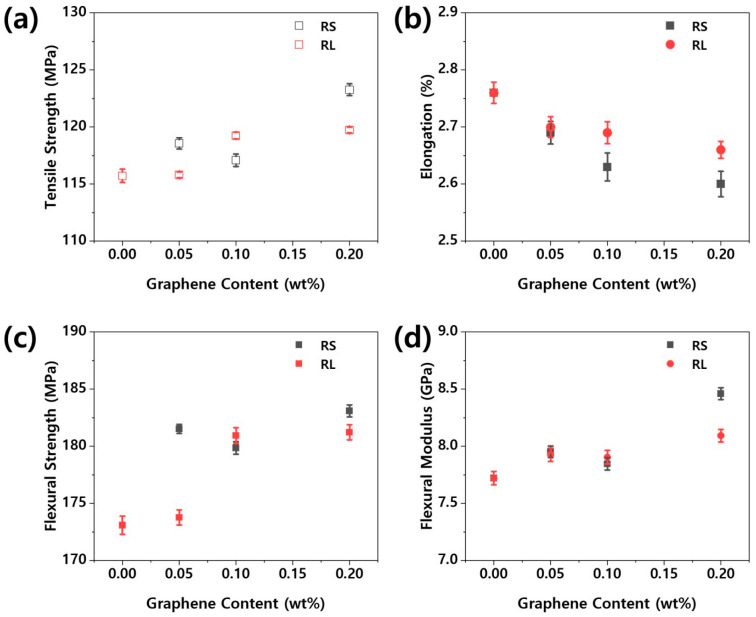

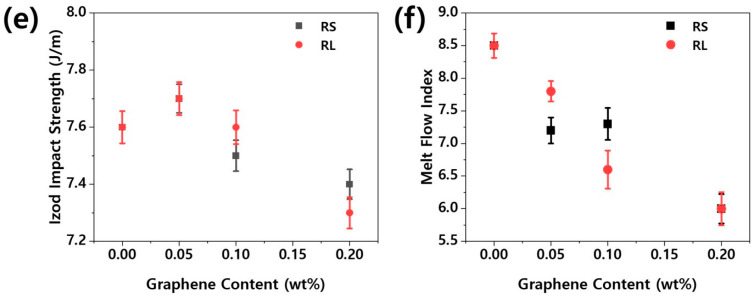
Mechanical properties of PBT/ASA nanocomposites with different graphene types and concentrations: (**a**) tensile strength, (**b**) elongation at break, (**c**) flexural strength, (**d**) flexural modulus, (**e**) Izod impact strength, and (**f**) melt flow.

Figure 5 illustrates the mechanical properties of the nanocomposites. The mechanical properties of nanocomposites are crucial for a wide range of applications. As shown in Figure 5, the tensile strength, flexural strength, and flexural modulus of the PBT/ASA–graphene nanocomposites were improved, likely due to enhanced compatibility between the nanofiller and the PBT/ASA matrix. In general, the mechanical properties of polymer nanocomposites are influenced by factors such as nanoparticle size, degree of dispersion, polymer–filler interface characteristics, and filler content [9]. The infiltration of nanofillers significantly affects the viscosity and melt flow behavior of the composites. At very low graphene content, the Izod impact strength was enhanced due to the effective infiltration of graphene into the PBT/ASA matrix. However, as the graphene content increased, the Izod impact strength showed a negative trend. Additionally, both the elongation at break and the melt flow index of the nanocomposites were found to decrease with increasing graphene content.

**Figure 6:**
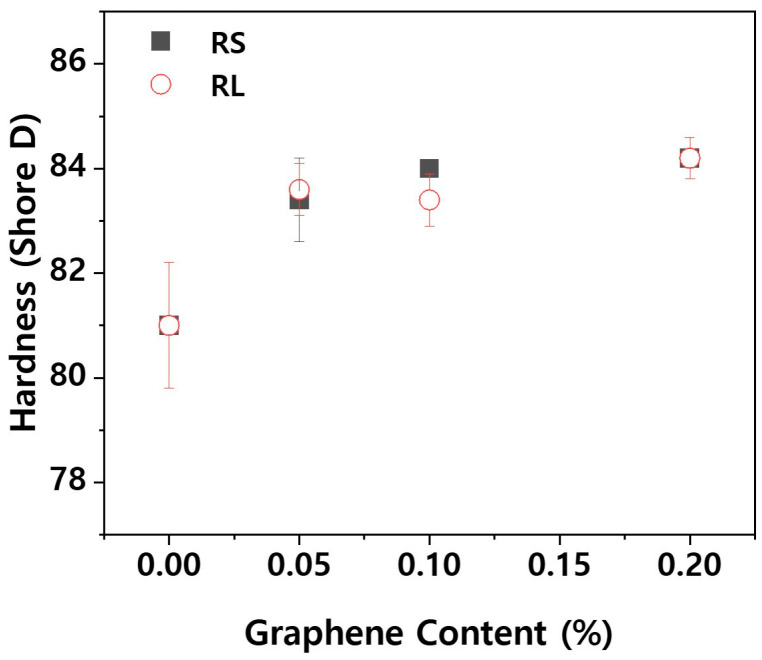
Shore D hardness of PBT/ASA nanocomposites with different graphene types and concentrations.

The lens holder of the projection module within a headlamp requires not only mechanical strength but also excellent tribological properties, as friction and wear are critical factors during operation. Wear particles generated from plastic components can contaminate the interior of the headlamp. One of the key properties influencing the wear characteristics of plastic materials is hardness. Figure 6 shows the Shore D hardness values for PBT/ASA nanocomposites. The hardness of plastics is measured using Shore A for softer materials and Shore D for harder materials. While the PBT/ASA-GF composite exhibited a hardness of 81.0, an increase in graphene content resulted in a corresponding rise in surface hardness. However, the difference in surface hardness between different types of graphene was negligible.

**Figure 7:**
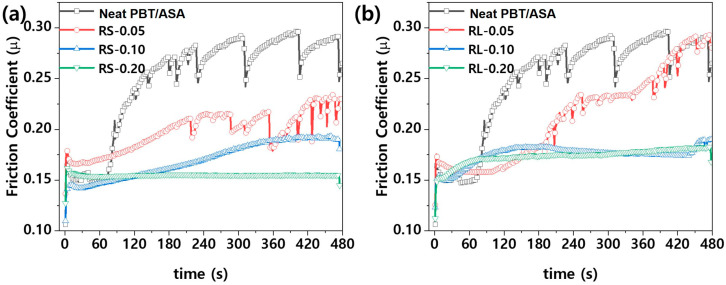
Friction coefficient of PBT/ASA nanocomposites with different graphene types and concentrations: (**a**) RS type and (**b**) RL type.

**Figure 8:**
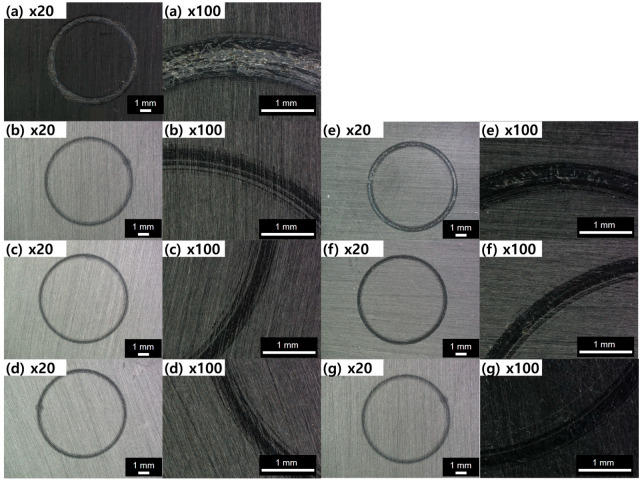
OM images of friction surface of the PBT/ASA nanocomposites with different graphene types and concentrations: (**a**) control, (**b**) RS-0.05, (**c**) RS-0.10, (**d**) RS-0.20, (**e**) RL-0.05, (**f**) RL-0.10, and (**g**) RL-0.20.

Figure 7 presents the friction coefficient graph of nanocomposites based on the type and content of graphene. As shown in Figure 7, the friction behavior of polymer nanocomposites shows two distinct trends. Initially, there is a significant increase in the friction coefficient due to the strong frictional force between the steel ball and the sample. As friction continues, the coefficient gradually decreases and stabilizes, which can be attributed to the smoothing of the contact surfaces. This behavior is common in composite materials [18,20]. The friction and wear phenomena observed are a combination of abrasive and adhesive wear, where polishing and smoothing are the primary damage mechanisms during friction. These mechanisms typically occur when a smooth and hard surface is in contact with another, causing polymer material to transfer to the harder surface and then be removed as wear debris [21,22]. For the PBT/ASA-GF reference sample, the friction coefficient initially increases and stabilizes until about 90 s, after which it sharply rises. The friction coefficient also fluctuates, showing periodic decreases followed by increases. The cause of this behavior can be inferred from the optical microscope images in Figure 8, which show the friction surface of the PBT/ASA nanocomposite. In Figure 8a, the friction area appears very rough. This instability in friction behavior is likely due to the removal of polymer material during initial friction, exposing the underlying glass fibers, which leads to uneven wear and higher friction. The graph in Figure 7 shows that when the graphene content is low, the solid lubrication properties of graphene result in a lower friction coefficient compared to the reference. However, as the friction time increases, the friction behavior becomes unstable, likely due to insufficient lubrication from the low graphene content. For example, as seen in Figure 8e, the RS-0.05 sample exhibits a rougher surface than the RS-0.05 sample in Figure 8b, correlating with its unstable friction behavior. When graphene content is sufficient, the solid lubrication effect is enhanced, leading to more stable friction behavior and a consistently lower friction coefficient.

**Figure 9:**
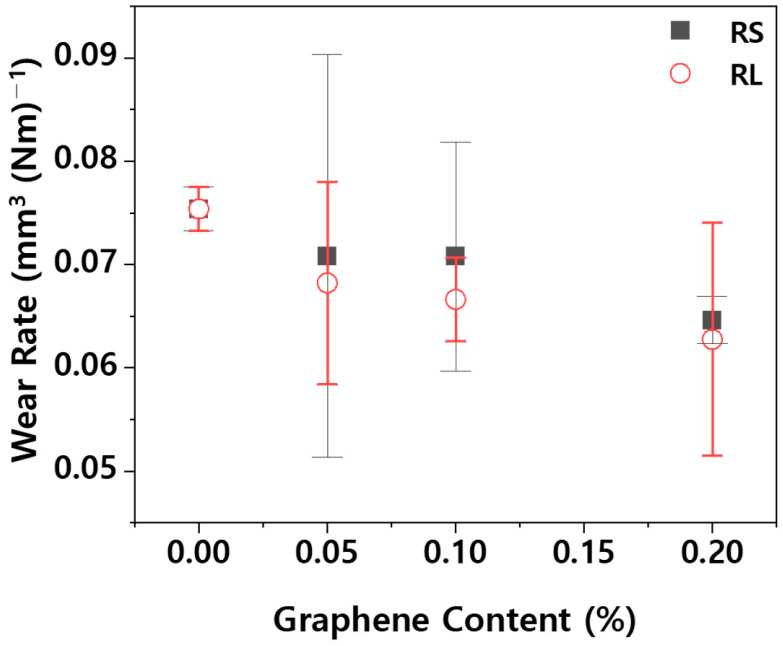
Wear rate of the PBT/ASA nanocomposites with different graphene types and concentrations.

Even though the friction coefficient is low, wear behavior cannot be fully explained by the friction coefficient alone, as wear rate can still be high [23]. Therefore, it is also necessary to examine the wear rate. Figure 9 shows the wear rate of PBT/ASA. The wear rate is influenced by the shape of the particles [24] and is closely related to surface hardness. As surface hardness increases, the wear rate tends to decrease. Specifically, as surface hardness improves, a corresponding reduction in wear rate is observed. For the PBT/ASA-GF reference sample, the wear rate is 7.54 × 10^−2^ mm^3^ N^−1^ m^−1^. However, the RS-0.20 sample showed a 14.3% reduction in wear rate, and the RL-0.20 sample demonstrated a 16.8% reduction. Based on the results of both the friction coefficient and wear rate, it can be concluded that the PBT/ASA nanocomposites exhibit improved wear resistance.

**Figure 10:**
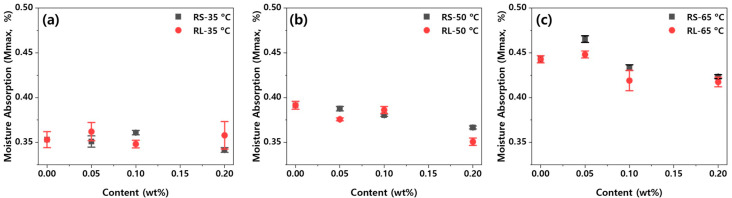
Maximum moisture absorption of the PBT/ASA nanocomposites at various temperatures plotted as a function of graphene content: (**a**) 35 °C, (**b**) 50 °C and (**c**) 65 °C.

Since PBT/ASA nanocomposites absorb moisture and release it depending on their use environment, the hygroscopic behavior of polymer composites used in components is crucial. Figure 10 shows the maximum moisture absorption as a function of graphene content and type at different ambient temperatures. As shown in Figure 10, the maximum moisture absorption tends to increase as the ambient temperature rises. At lower temperatures, the differences in maximum moisture absorption between the various types and contents of graphene are minimal. However, at higher ambient temperatures, an increase in graphene content results in a noticeable decrease in maximum moisture absorption. This indicates that graphene effectively blocks moisture diffusion pathways, demonstrating its efficacy in reducing moisture absorption.

**Figure 11:**
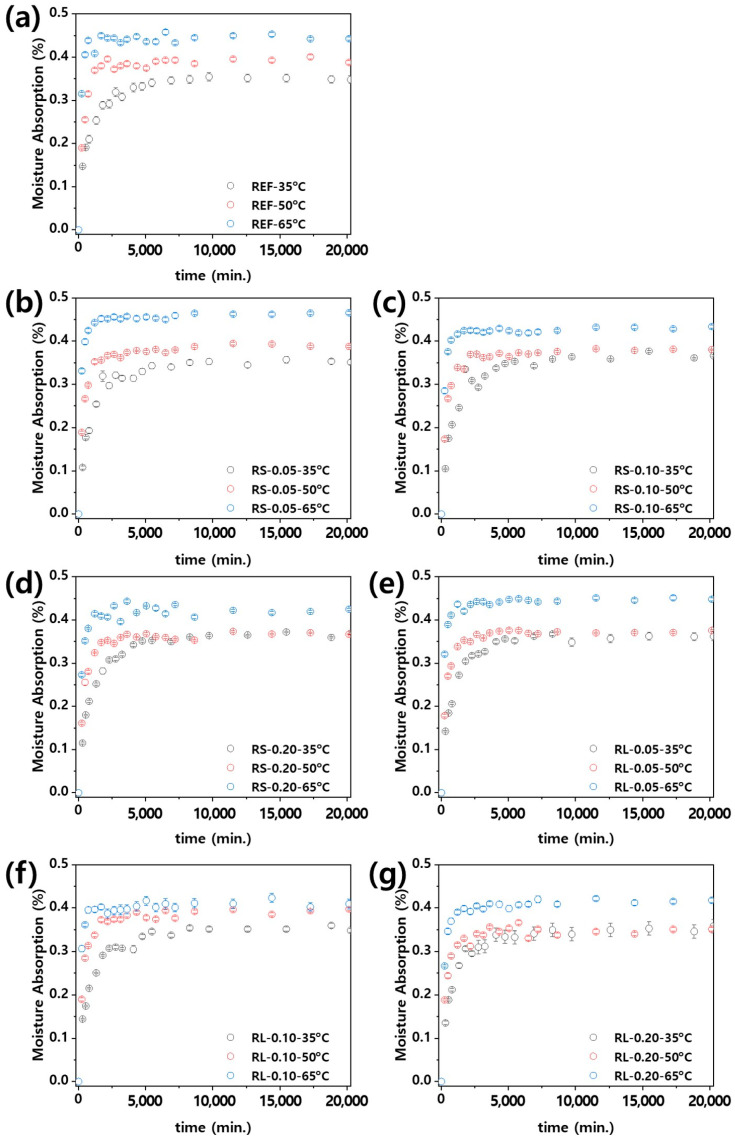
Moisture absorption of PBT/ASA nanocomposites at various temperatures plotted as function of time: (**a**) reference, (**b**) RS-0.05, (**c**) RS-0.10, (**d**) RS-0.20, (**e**) RL-0.05, (**f**) RL-0.10, and (**g**) RL-0.20.

Figure 11 presents a graph showing the moisture absorption rate of the nanocomposites over time, calculated according to Equation (3). Studies on the moisture absorption behavior of polymers often use diffusion-controlled equations, such as Fick’s second law, to model various aspects of the process [11,12,13,14,15]. Moisture absorption is described as the cumulative uptake of moisture in the material as a function of time, represented by the diffusion coefficient (D). Crank et al. [25] developed a simplified mathematical solution to Fick’s second law based on trigonometric functions, which applies to a large flat plate with thickness *h*. The amount of absorbed water at any given time *t*, Mt (%), can be calculated using Equation (3):

(3) $$\frac{M_{t}}{M_{Max}}=1-\frac{8}{\pi^{2}}\sum_{n=0}^{n=\infty }\frac{1}{{\left(2n+1\right)}^{2}}exp(\frac{-D\pi^{2}{\left(2n+1\right)}^{2}}{h^{2}}t)$$

where $M_{max}$ represents the maximum moisture absorption (%), D is the diffusion coefficient (m^2^ min^−1^), and h is the thickness of the sample (m). As shown in Figure 11, the moisture absorption of the nanocomposites increases over time until it reaches an equilibrium state after absorbing a certain amount of moisture. When considering the influence of ambient temperature, the time required to reach equilibrium decreases as the ambient temperature increases. Figures S1–S3 present the moisture absorption graphs of PBT/ASA nanocomposites at different ambient temperatures, plotted using Equation (3) for curve fitting. As described earlier, each nanocomposite exhibits moisture absorption behavior consistent with the diffusion-controlled equation. Additionally, the adjusted coefficient of determination (adj. $R^{2}$) for the curve-fitting method ranges from 0.90 to 0.99, indicating a very high level of accuracy. This fitting allows for the extraction of the diffusion coefficients.

**Figure 12:**
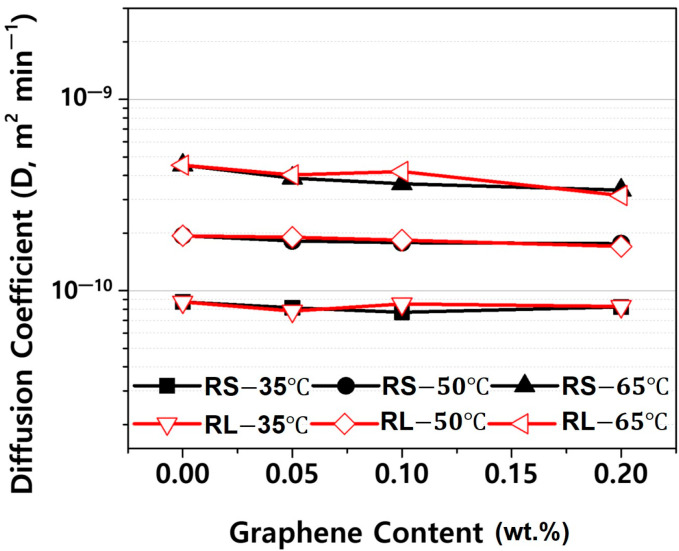
Diffusion coefficient in absorption processes of PBT/ASA nanocomposites at various temperatures plotted as a function of graphene content.

Figure 12 presents the diffusion coefficients derived from the fitting curves in Figures S1–S3. As shown in Figure 12, the diffusion coefficient decreases slightly as the graphene content increases. This is likely due to the increased amount of graphene sheets, which act as barriers, reducing the rate of moisture absorption [9,10]. However, the lateral size of graphene does not appear to significantly affect the diffusion coefficient at the same graphene content. This can be attributed to the fact that lateral size alone does not dictate diffusion pathways; rather, overall dispersion, alignment, and interfacial adhesion of graphene within the matrix are more critical in influencing moisture diffusion. Therefore, similar diffusion coefficients are observed despite differences in lateral size when graphene content is constant. Meanwhile, the diffusion coefficient increases with rising ambient temperature, indicating that the temperature has a much larger effect on the diffusion coefficient than the graphene additive. This is because the diffusion of moisture in plastics is primarily an energy activation process, and the diffusion coefficient follows an Arrhenius equation, which exhibits exponential temperature dependence alongside activation energy [11,12,13,14].

**Figure 13:**
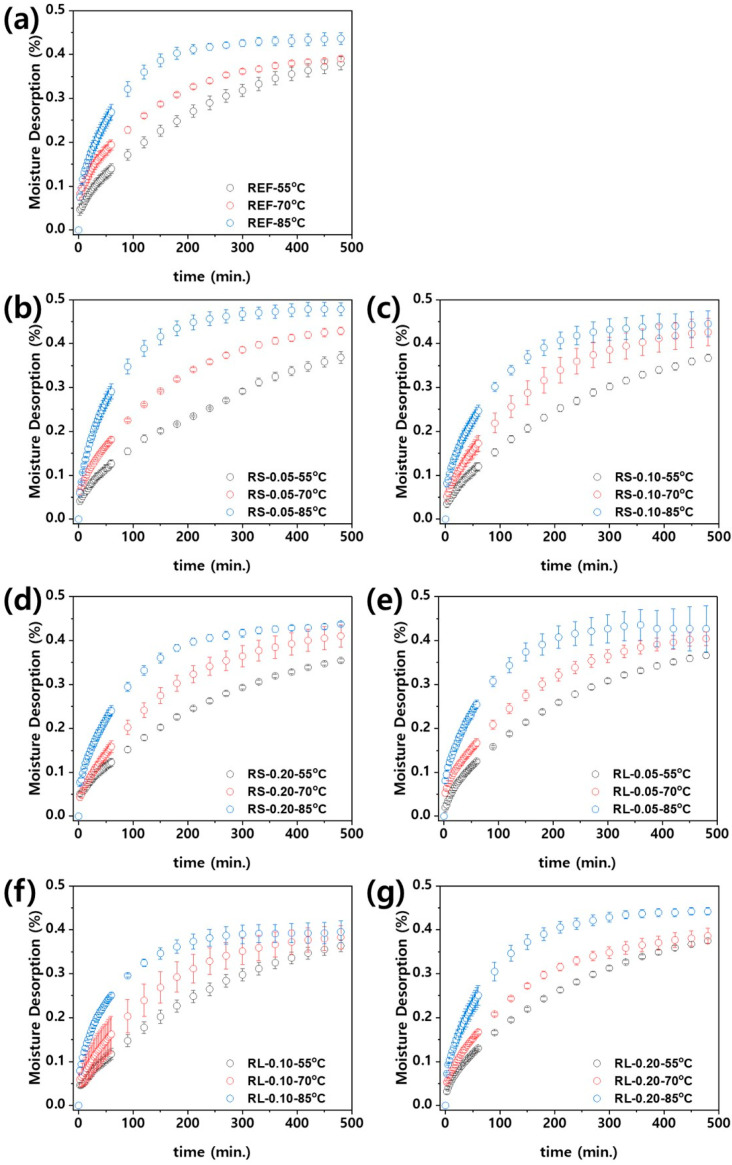
Moisture desorption of PBT/ASA nanocomposites at various temperatures plotted as function of time: (**a**) reference, (**b**) RS-0.05, (**c**) RS-0.10, (**d**) RS-0.20, (**e**) RL-0.05, (**f**) RL-0.10, and (**g**) RL-0.20.

Figure 13 presents the moisture desorption of the nanocomposites over time, calculated according to Equation (2). Similar to the moisture absorption behavior, the desorption process also follows a diffusion-governing equation. As shown in Figure 13, the desorption of the nanocomposites increases over time until all the moisture inside the polymer is released, eventually reaching an equilibrium state. When examining the influence of ambient temperature, it becomes clear that the time required to reach equilibrium decreases as the ambient temperature increases. Figures S4–S6 show the moisture desorption curves of PBT/ASA nanocomposites at different ambient temperatures, plotted using Equation (2) for curve fitting. As with hygroscopic behavior, it can be seen that each nanocomposite material exhibits hygroscopic behavior according to the diffusion-governing equation. Furthermore, the curve-fitting method shows a very high level of accuracy, with adjusted coefficients of determination (adj. R^2^) ranging from 0.90 to 0.99. This high accuracy allows for the calculation of the diffusion coefficients.

**Figure 14:**
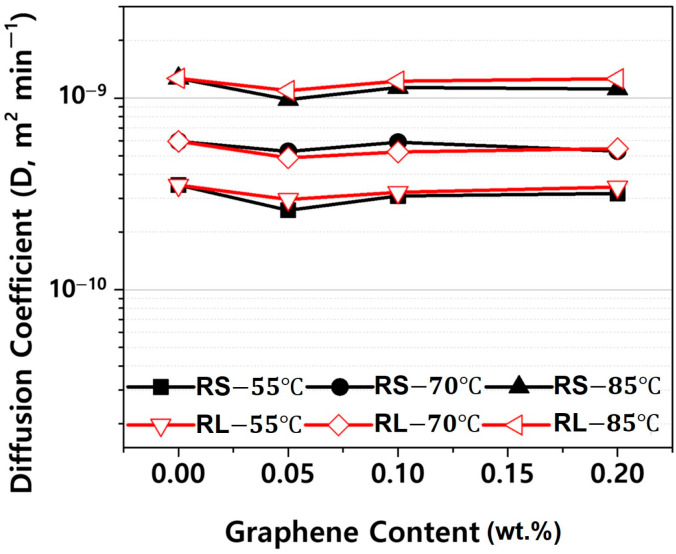
Diffusion coefficient in desorption processes of PBT/ASA nanocomposites at various temperatures plotted as a function of graphene content.

When comparing Figure 12 and Figure 14, it is evident that the diffusion coefficient during the desorption process is higher than that during the absorption process. In a closed system where no external moisture is supplied, polymers undergo a reversible reaction, simultaneously absorbing and releasing moisture. If the diffusion coefficient for desorption is higher than that for absorption, desorption becomes the dominant process. Therefore, in sealed environments like headlamps, the internal absolute humidity gradually increases, leading to condensation on the inner surface of the outer lens due to temperature differences with the outside. The variation in the difference between absorption and desorption diffusion coefficients with respect to the nanofiller content was found to be negligible.

**Figure 15:**
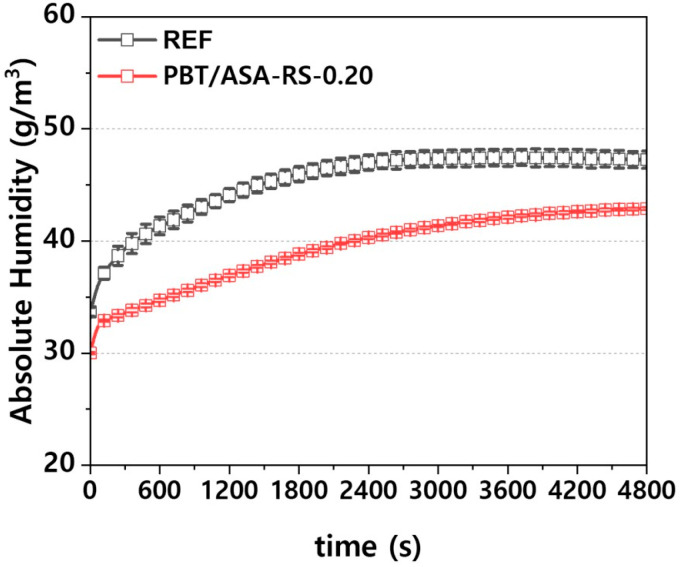
Absolute humidity behavior of PBT/ASA nanocomposites inside the headlamp.

To verify the moisture reduction effect of the PBT/ASA nanocomposites, headlamps were manufactured, and the internal absolute humidity was measured. Measurements were taken at five different points, and the average values are presented. As shown in Figure 15, the error at each measurement point is minimal. It can be observed from the figure that as the lamp operation time increases, the absolute humidity increases because of moisture released from within the polymer. However, the nanocomposite’s barrier properties cause the rate of moisture release to be lower compared to conventional composites. Additionally, during headlamp preconditioning, the nanocomposites absorbed less external moisture than conventional composites, resulting in a reduced amount of released moisture during desorption.

As previously discussed, the moisture absorption and desorption behavior of polymer materials used in sealed components, such as headlamps, can lead to issues like fogging. After injection molding, polymer parts undergo cooling, storage, transportation, pre-assembly staging, and assembly, during which they absorb moisture from the air. While thorough drying before assembly can minimize moisture content, completely eliminating it is challenging and would lead to increased time and costs, making it impractical for real-world production lines. Therefore, understanding the moisture absorption and desorption characteristics of the materials used is essential to address these issues effectively through optimized part design.

## 4. Conclusions

In this study, the low-moisture absorption behavior of PBT/ASA composites used in headlamp lens holders was investigated. A graphene-based nanofiller was synthesized to achieve low-moisture absorption properties, and PBT/ASA nanocomposites were developed. The inclusion of graphene improved the tensile and flexural strength of the PBT/ASA nanocomposites, though it led to reduced flowability and elongation. Additionally, surface hardness increased, enhancing wear resistance. Examination of the moisture absorption and desorption behavior of the PBT/ASA nanocomposites revealed improved performance compared to commercial PBT/ASA composites, suggesting a potential reduction in headlamp fogging. The internal absolute humidity of the headlamps using the developed material was measured, showing a decrease, thereby confirming the improved moisture absorption and desorption characteristics of the PBT/ASA nanocomposites.

## Data Availability

All data used during the study appear in the submitted article.

## Supplementary Materials

The following supporting information can be downloaded at: https://www.mdpi.com/article/10.3390/polym16223149/s1, Figure S1: Moisture absorption of PBT/ASA nanocomposites at various graphene content and 35 °C plotted as a function of time: (a) reference, (b) RS-0.05, (c) RS-0.10, (d) RS-0.20, (e) RL-0.05, (f) RL-0.10, and (g) RL-0.20; Figure S2: Moisture absorption of PBT/ASA nanocomposites at various graphene content and 50 °C plotted as a function of time: (a) reference, (b) RS-0.05, (c) RS-0.10, (d) RS-0.20, (e) RL-0.05, (f) RL-0.10, and (g) RL-0.20; Figure S3: Moisture absorption of PBT/ASA nanocomposites at various graphene content and 65 °C plotted as a function of time: (a) reference, (b) RS-0.05, (c) RS-0.10, (d) RS-0.20, (e) RL-0.05, (f) RL-0.10, and (g) RL-0.20; Figure S4: Moisture desorption of PBT/ASA nanocomposites at various graphene content and 55 °C plotted as a function of time: (a) reference, (b) RS-0.05, (c) RS-0.10, (d) RS-0.20, (e) RL-0.05, (f) RL-0.10, and (g) RL-0.20; Figure S5: Moisture desorption of PBT/ASA nanocomposites at various graphene content and 70 °C plotted as a function of time: (a) reference, (b) RS-0.05, (c) RS-0.10, (d) RS-0.20, (e) RL-0.05, (f) RL-0.10, and (g) RL-0.20; Figure S6: Moisture desorption of PBT/ASA nanocomposites at various graphene content and 85 °C plotted as a function of time: (a) reference, (b) RS-0.05, (c) RS-0.10, (d) RS-0.20, (e) RL-0.05, (f) RL-0.10, and (g) RL-0.20.
