# Supplementary material for: Tribological and Hygroscopic Behavior of Polybutylene Terephthalate/Acrylonitrile Styrene Acrylate (PBT/ASA) Nanocomposites with Graphene Nanofiller
Source: Polymers (Basel). 2024 Nov 12;16(22):3149. doi: 10.3390/polym16223149 (PMC11598128; doi:10.3390/polym16223149)
Supplement: Supplementary file 1 [file polymers-16-03149-s001.zip › polymers-3300643-supplementary.pdf]

Supplementary information

Tribological and Hygroscopic Behavior of Polybutylene Terephthalate/Acrylonitrile Styrene Acrylate (PBT/ASA) Nanocomposites with Graphene Nanofiller

Pyoung-Chan Lee[1], Seo-Hwa Hong[1], Ji Taek Oh[2], Donghyeok Shin[3], Jae-Uk Jung[4], Youn Ki Ko[1], Jin Uk Ha[1], Myeong-Gi Kim[2,*], and Beom-Gon Cho[5,*]

[1] Chassis & Materials Research Laboratory, Korea Automotive Technology Institute, 303 Pungse-ro, Pungse-myeon, Dongnam-gu, Cheonan-si, Chungcheongnam-do 31214, Republic of Korea; pclee@katech.re.kr (P.-C.L.); shhong1@katech.re.kr (S.H.H); ykko@katech.re.kr (Y.K.K); juha@katech.re.kr (J.U.H);
[2] R&D Center, BESTGRAPHENE Co., Ltd, Yeoju-si, Gyeonggi-do 12616, Republic of Korea; mgkim@best-graphene.com (M.-G.K); ojt3413@best-graphene.com (J.T.O)
[3] R&D Center, WOOSUNG Advanced Materials Co., Ltd., Mungyeong-si, Gyeongbuk 36990, Republic of Korea;
sdh@metapoly.co.kr (D.H.S.)
[4] R&D Planning Department, Nifco Korea Co., Ltd., Asan-si, Chungnam 31409, Republic of Korea
jujung@nifco.co.kr (J.-U.J)
[5] Department of Polymer Science and Engineering, Kumoh National Institute of Technology, 61 Daehak-ro, Gumi, Gyeongbuk 39177, Republic of Korea
bgcho@kumoh.ac.kr (B.-G.C)

*Correspondence: mgkim@best-graphene.com (M.-G.K) Tel.: (+82-31-883+8858); bgcho@kumoh.ac.kr (B.-G.C) Tel.: (+82-54-478-7684)

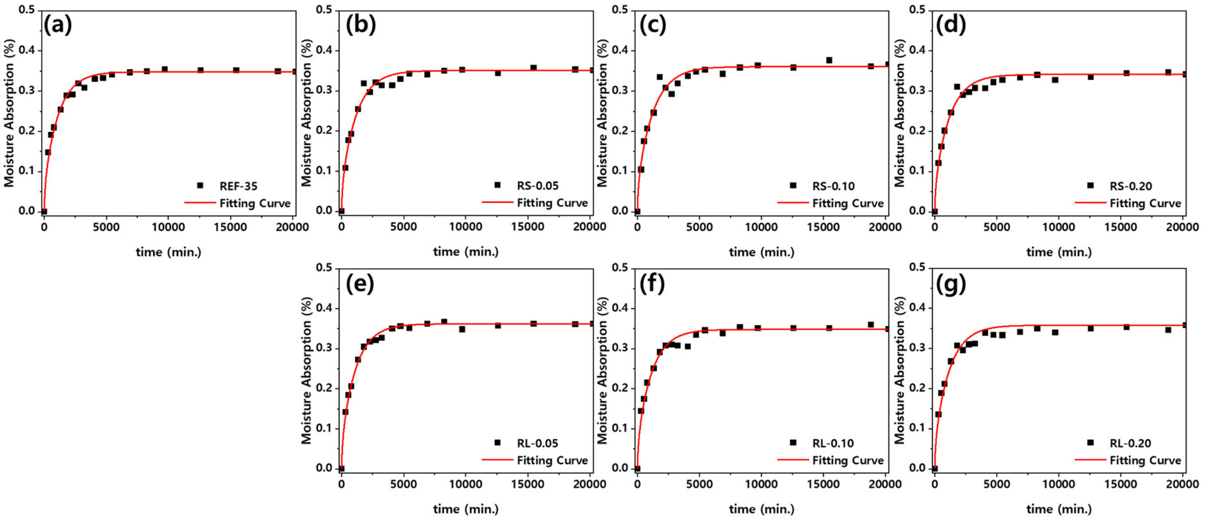

Figure S1. Moisture absorption of PBT/ASA nanocomposites at various graphene content and 35 ºC plotted as a function of time: (a) reference, (b) RS-0.05, (c) RS-0.10, (d) RS-0.20, (e) RL-0.05, (f) RL-0.10, and (g) RL-0.20.

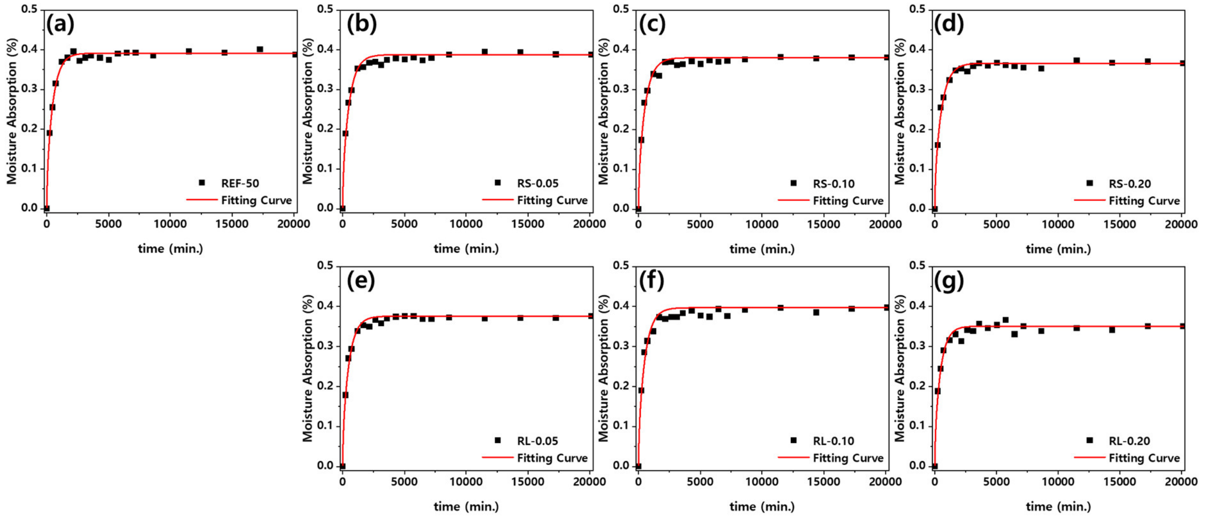

Figure S2. Moisture absorption of PBT/ASA nanocomposites at various graphene content and 50 ºC plotted as a function of time: (a) reference, (b) RS-0.05, (c) RS-0.10, (d) RS-0.20, (e) RL-0.05, (f) RL-0.10, and (g) RL-0.20.

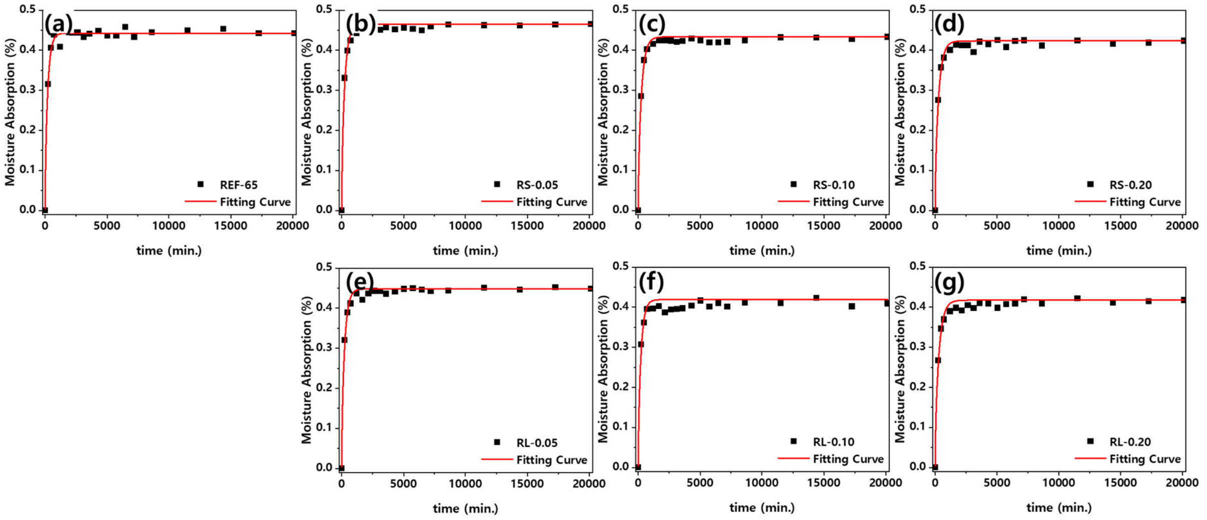

Figure S3. Moisture absorption of PBT/ASA nanocomposites at various graphene content and 65 ºC plotted as a function of time: (a) reference, (b) RS-0.05, (c) RS-0.10, (d) RS-0.20, (e) RL-0.05, (f) RL-0.10, and (g) RL-0.20.

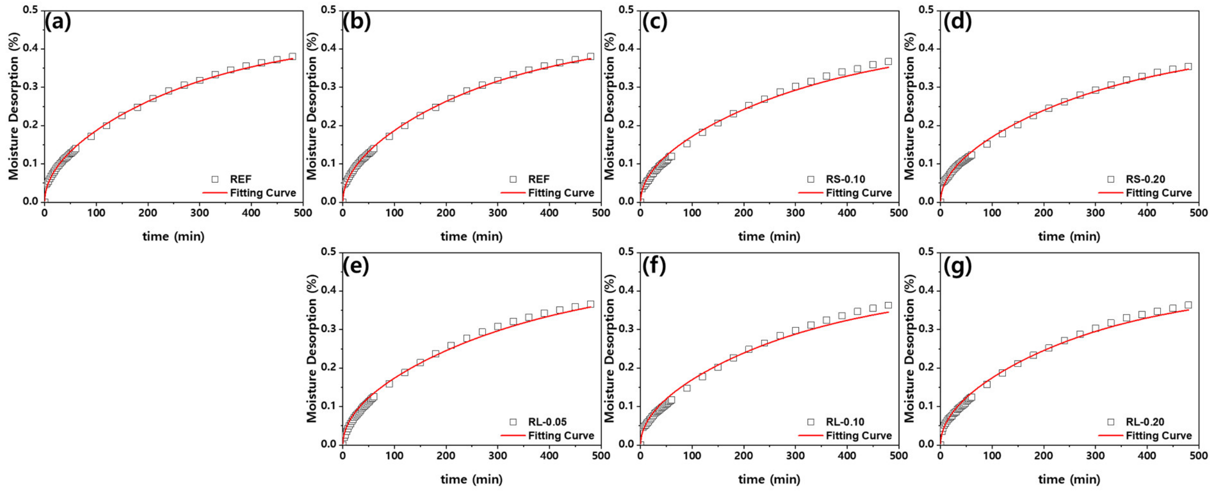

Figure S4. Moisture desorption of PBT/ASA nanocomposites at various graphene content and 55 ºC plotted as a function of time: (a) reference, (b) RS-0.05, (c) RS-0.10, (d) RS-0.20, (e) RL-0.05, (f) RL-0.10, and (g) RL-0.20.

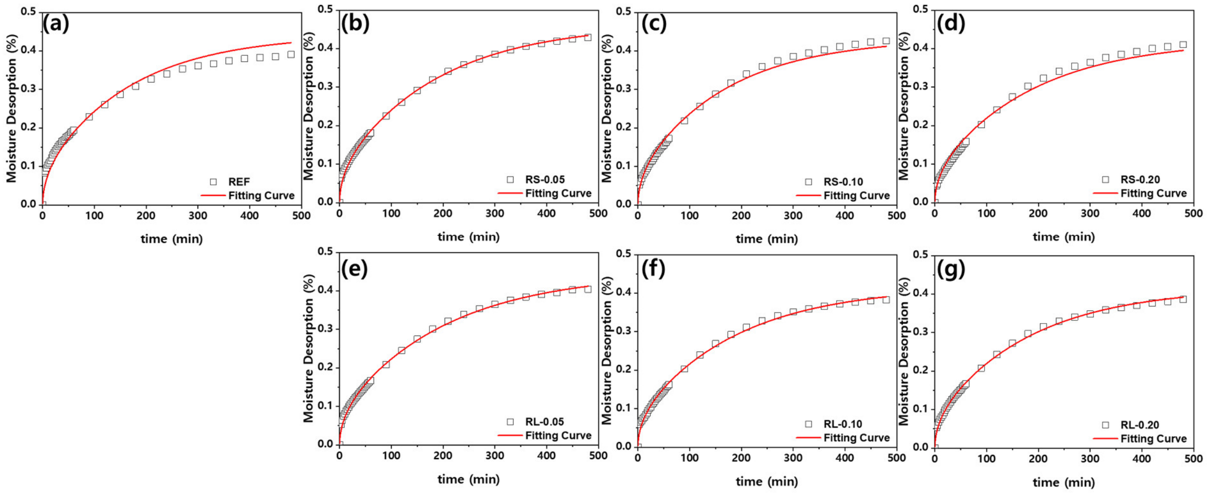

Figure S5. Moisture desorption of PBT/ASA nanocomposites at various graphene content and 70 ºC plotted as a function of time: (a) reference, (b) RS-0.05, (c) RS-0.10, (d) RS-0.20, (e) RL-0.05, (f) RL-0.10, and (g) RL-0.20.

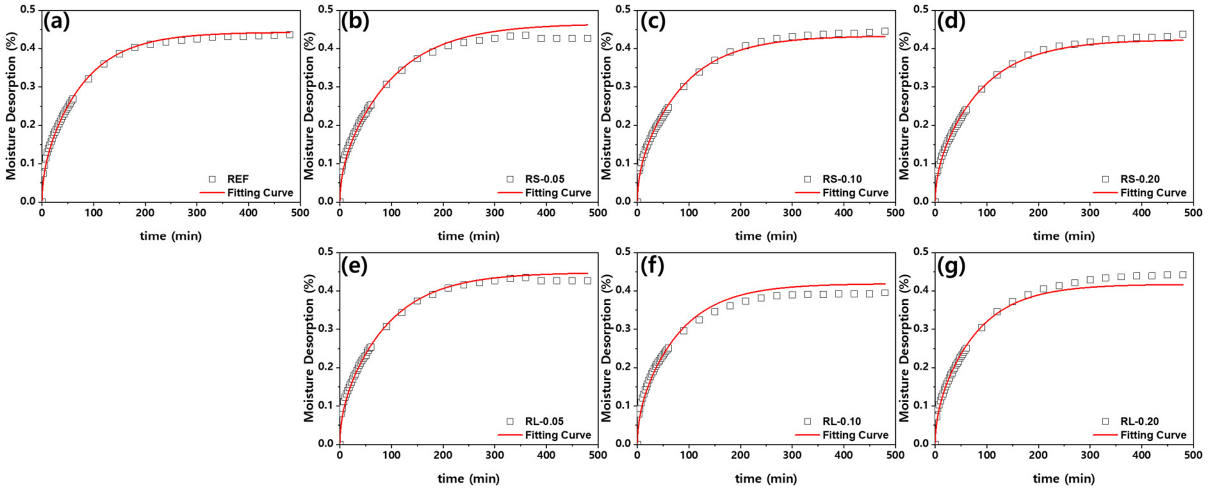

Figure S6. Moisture desorption of PBT/ASA nanocomposites at various graphene content and 85 ºC plotted as a function of time: (a) reference, (b) RS-0.05, (c) RS-0.10, (d) RS-0.20, (e) RL-0.05, (f) RL-0.10, and (g) RL-0.20.
